# Integrative Genomic Analysis Reveals Extended Germline Homozygosity with Lung Cancer Risk in the PLCO Cohort

**DOI:** 10.1371/journal.pone.0031975

**Published:** 2012-02-27

**Authors:** Mohammed S. Orloff, Li Zhang, Gurkan Bebek, Charis Eng

**Affiliations:** 1 Genomic Medicine Institute, Lerner Research Institute, Cleveland Clinic, Cleveland, Ohio, United States of America; 2 Department of Quantitative Health Sciences, Lerner Research Institute, Cleveland, Ohio, United States of America; 3 Taussig Cancer Institute, Cleveland, Ohio, United States of America; 4 Stanley Shalom Zielony Institute of Nursing Excellence, Cleveland Clinic, Cleveland, Ohio, United States of America; 5 Department of Genetics, Case Western Reserve University School of Medicine, Cleveland, Ohio, United States of America; 6 CASE Comprehensive Cancer Center, Case Western Reserve University School of Medicine, Cleveland, Ohio, United States of America; Ohio State University Medical Center, United States of America

## Abstract

Susceptibility to common cancers is multigenic resulting from low-to-high penetrance predisposition-factors and environmental exposure. Genomic studies suggest germline homozygosity as a novel low-penetrance factor contributing to common cancers. We hypothesized that long homozygous regions (tracts-of-homozygosity [TOH]) harbor tobacco-dependent and independent lung-cancer predisposition (or protection) genes. We performed i*n silico* genome-wide SNP-array-based analysis of lung-cancer patients of European-ancestry from the PLCO screening-trial cohort to identify TOH regions amongst 788 cancer-cases and 830 ancestry-matched controls. Association analyses was then performed between presence of lung cancer and common(c)TOHs (operationally defined as 10 or more subjects sharing ≥100 identical homozygous calls), aTOHs (allelically-matched groups within a cTOH), demographics and tobacco-exposure. Finally, integration of significant c/aTOH with transcriptome was performed to functionally-map lung-cancer risk-genes. After controlling for demographics and smoking, we identified 7 cTOHs and 5 aTOHs associated with lung cancer (adjusted p<0.01). Three cTOHs were over-represented in cases over controls (OR = 1.75–2.06, p = 0.007–0.001), whereas 4 were under-represented (OR = 0.28–0.69, p = 0.006–0.001). Interaction between smoking status and cTOH3/aTOH2 (2p16.3–2p16.1) was observed (adjusted p<0.03). The remaining significant aTOHs have ORs 0.23–0.50 (p = 0.004–0.006) and 2.95–3.97 (p = 0.008–0.001). After integrating significant cTOH/aTOHs with publicly-available lung-cancer transcriptome datasets followed by filtering based on lung cancer and its relevant pathways revealed 9 putative predisposing genes (p<0.0001). In conclusion, differentially-distributed cTOH/aTOH genomic variants between cases and controls harbor sets of plausible differentially-expressed genes accounting for the complexity of lung-cancer predisposition.

## Introduction

There are two main histologic groupings in lung cancer, small cell lung cancer (SCLC) and non-small cell lung cancer (NSCLC). The latter includes adenocarcinoma (AC) and squamous cell carcinoma (SCC), along with less common subtypes. It has been widely accepted that an average of 5–10% of all malignancies are caused by high penetrance predisposition genes [1]–[3]. For example, there are 10 high penetrance genes, including *BRCA1/2* and *PTEN*, accounting for ∼10% of all breast cancers [3]. While aerodigestive tract cancers are believed to be a rare part of the neoplastic spectrum of *BRCA2*, no other high penetrance lung-cancer-predisposition gene has been identified, and until recently, lung cancer has been attributed almost entirely to environmental exposure, chiefly tobacco. In the last few years, however, it has become obvious that a greater, but variable, proportion of all malignancies have a genomic component, conferring weaker predisposition (low penetrance). Eg, a genome-wide association study (GWAS) demonstrated specific single nucleotide polymorphims (SNPs) associated with risk of AC in smokers and never-smokers [4]. To date, NSCLC, especially AC-associated genomic-loci have been identified in 15q25, 5p15, and 6p21 [5]–[10]. Analysis of the effect of smoking on lung-cancer risk showed that smoking does not entirely explain the risk of developing lung cancers and that residual genomic-factors interacting with smoking are likely [4]. Genomic variants, such as the associated SNPs, cannot fully explain the heterogeneity associated with the histologic subtypes either [11], [12]. The evidence to date suggests the need to find other types of genomic variation that can explain the relatively large remaining risk associated with lung carcinomas.

In animal husbandry and animal-model experimentation, in-breeding which results in increasing homozygous loci is well recognized to result in increased incidence of various disorders, including increasing tumor incidence [13]. In humans, germline homozygosity as a genomic factor associated with disease-risk is a relatively recent concept. Eg, germline homozygosity, a type of genomic variation, has been shown to be associated with an increased risk of human cervical cancer. Identification of homozygous loci as risk factors may help target heightened cervical screening for high-risk women [14]–[18]. Relatedly, a relatively recent study uncovered a significantly higher frequency of germline homozygosity in a series of unrelated white individuals with invasive breast carcinomas, prostate carcinomas and head neck squamous cell caricinomas by genome-wide microsatellite genotyping [19]. This association was validated in a study of AC cases and matched-controls that were genotyped with denser SNP-based arrays (Illumina HumanHap550v3_B array), thus supporting the high likelihood of identifying homozygous genotypes that are associated with a broad variety of common solid tumors [19]. This study observed that homozygosity from both microsatallite- and SNP-based analyses showed specific, shared loci of homozygosity for all the three tumor types studied. In addition, there were also highly homozygous loci that are specific to each of the tumor types. Independently, Bacolod and colleagues [20] found that long tracts of homozygosity (TOH), operationally defined as spanning at least 4 Mb, were over-represented in colorectal cancer patients over controls.

Here, we hypothesized that germline regional-homozygosity involving specific chromosomal loci is a novel genomic factor contributing to low- to-moderate penetrance predisposition to (or protection from) lung cancer. Instead of identifying single genes, our hypothesis takes into account subsets of genes within these regions, which are differentially expressed to lend complex predisposition to lung cancer. We sought to address this hypothesis by systematically integrating data from differentially represented TOH regions with genome-wide expression data to localize regional lung-cancer predisposition loci.

## Methods

### Acquisition of Genotype Data from dbGAP

Genotypes were obtained from the Prostate, Lung, Colorectal and Ovarian cancer screening trial (PLCO) where the lung cohort was prospectively screened with chest X-rays [21]. Subjects were all self-identified as white, and comprise ancestry-matched cases and controls [21] based on principle-component analyses using both SNPs unlinked to lung cancer and their ancestry-informed SNP's, as described by Patterson et al [22]. Consistently, the CEPH (Centre d'Etude du Polymorphisme Humain) from Utah (CEU) HapMap controls cluster with this population, re-confirming northern and western European origin [23].

We followed the standard quality control (QC) procedure used in the original study [4]. Samples were screened and selected only if they had a minimum 95% successful genotype call rate. SNPs with minor allele frequencies (MAF) <5%, departures from Hardy-Weinberg equilibrium (at p<0.01) and ≥5% missingness per SNP, were excluded from further analyses. After QC filtering, we had 1618 subjects (788 cases and 830 ancestry matched controls) with mean age categories of 1.63 (5 categories defined in Table S1), comprising 967 males and 651 females, including 156 nonsmokers, 703 previous smokers and 759 current smokers (Table S1); and an average 526,826 (514,355 autosomal) SNPs (93.8%)/subject. Table S1 shows the association analysis based on a logistic model with age, gender and smoking status (never smoked, previous smoking and current smoking) as covariates after excluding the potential genetic effects. It is important to note that the proportion of current smokers was about half the rate of active smokers in the US general population. It was noted that compliance was the lowest in the current smokers whereas the previous smokers were the most compliant.

### Quantifying Tracts of Homozygosity and Comparing Frequencies in Cancer Cases and Controls

#### Identifying tracts of homozygosity (TOH) and common TOH (cTOH) region

We extended the module of Runs of Homozygosity in the GoldenHelix software [24] to identify TOHs [an in-house software (Zhang et al, unpublished)]. Next, data from all subjects were examined to determine whether a minimum number of individuals share a TOH call at a given position. To identify statistical differences between TOHs within a case-control design, we only retained those TOHs in which 10 or more subjects share 100 identical homozygous calls, which we operationally define as a common TOH (cTOH). There are 333,861 SNPs with 10 or more TOH calls across the entire series, representing 65% of the original pool of SNPs.

#### Detection of cTOHs associated with lung cancer

We then pursued testing for association between cTOH and lung-cancer cases. By considering each cTOH as a genomic variant, a genome-wide case-control analysis was conducted for each cTOH, where a cTOH was viewed as a binary variable based on the presence or absence of a cTOH. Using each TOH (containing multiple SNPs that are in linkage disequilibrium) as a variable will considerably reduce the number of tests to be performed and boost the power of the association analysis. The traditional single SNP-association studies require at least 610 000 (up to 3 million if more SNPs are used) tests if a traditional GWAS was done. A logistic model was fitted for each cTOH by considering disease status as the outcome and the cTOH as the predictor. Other covariates included in the model were age, sex and smoking status. P-values were obtained by Wald tests and OR (95% CI) were calculated through coefficient estimates of the fitted logistic model. To detect interactions between cTOH and smoking status, and cTOH and age, a logistic model with two extra interaction terms was fitted for each cTOH. The P-value of interaction was obtained by F-test. To minimize chances of false positive findings, cTOHs are considered statistically significant if their p<0.01 [24]. Furthermore, the q-value approach [25], that is based on the concept of the false discovery rate, was used as an exploratory guide for which the variants called can be investigated further.

#### Investigating allelically-matched groupings within a cTOH (aTOH)

As noted above, a cTOH is operationally defined by a minimum number of loci that are homozygous and minimum number of subjects sharing the cTOH, but not qualitative matching of nucleotides. Within the cTOH, TOH segments were then compared pair-wise and an allelic match is declared if at least 0.95 of jointly non-missing, jointly homozygous sites are identical. These allelic matching groups of TOHs within a cTOH are termed ‘allelic’TOH (aTOH). The characterization and scanning of these aTOHs was performed using our customized software *cag-TOH* (unpublished software), similar to the allelic-matching procedure in PLINK [26].

#### Detection of aTOHs associated with lung cancer cases

The aTOH as genomic variant was then used for association analysis within a case-control framework. To retain the power of the statistical analysis, we only focused on the aTOHs which are present in at least 5 cases and 5 controls. For each aTOH, we applied a logistic model with disease status as the outcome and aTOH as a predictor with age, sex and smoking status as covariates. Similar to cTOH above, the aTOHs with p<0.01 by Wald-test are declared significantly associated with lung cancer. We also applied the q-value approach [25].

### Integrating Genetic Information from Significant c/aTOH Regions with Publicly Available Expression Array Dataset

Data were obtained from a publicly available [27] gene-expression dataset of 107 fresh frozen tissue samples of AC (58 tumor and 49 non-tumor tissues from 20 never smokers, 26 former smokers, and 28 current smokers) downloaded from the Gene Expression Omnibus (GSE10072), from the Environment And Genetics in Lung cancer Etiology (EAGLE) study (http://dceg.cancer.gov/eagle). The criteria used to select this particular array dataset provide not only minimal bias, but physiologically relevant data. We followed the universal standard that specific selection criteria and QC's are in place before using publicly available datasets (e.g. expression array) for cross-platform integration purposes. Therefore, we ensured that the lung cancers in the expression array datasets belong to patients who are similar to those patients who were genotyped and subjected to TOH analysis. For example, patients utilized in both expression array and TOH analysis represent two different subsets of one much larger study cohort. This by itself is a major strength of this cross platform integration process because patients in the two datasets were subjected to the same inclusion/selection criteria; these individuals have been exposed to similar environmental or treatment conditions; most importantly, ancestral background of the “expression array dataset” patients were similar to those who were genotyped for TOH analysis; and the patients are of the same age ranges, i.e. 55–60 yrs. After QC, we normalized the expression profiles of the samples using the Robust Multichip Average (RMA) method, similar to how the same expression array data were originally processed [28]. The raw probes are mapped to their corresponding genes, and multiple probes corresponding to the same gene were averaged. The significant cTOH regions were first extended 250 kb in each direction, and genes within these regions were identified (259 genes). The number of genes included in the region increases linearly as the flanking regions are extended, but is also dependent on the region being interrogated (i.e., if a gene rich or gene poor region). If it returned >1000 genes (which we did not observe in our analyses here), we would have simply used LD to capture the block of cTOH or aTOH. The microarray expression profiles of 153 of the 259 cTOH-genes were found on the expression array. Subsequently, we evaluated on an *a priori* basis differences in expression profiles of these 153 genes using individual univariate logistic regression with Bonferroni correction applied for statistical significance calculations (data not shown). Expression profiles of the significant genes from univariate analysis (p<0.01) and within the +/−250 kb region of c/aTOH region were subjected to unsupervised hierarchical clustering [29] using Matlab®.

### Prioritization of Candidate Genes

After integrating significant c/aTOH regions with the expression array dataset, we determined the risk associated with differential expression of genes with c/aTOHs stratified by smoking status. Genes that showed differential expression profiles significant at p<0.0001 in the ever- and never-smoking strata were then subjected to a text mining approach to help filter from relevant information generated from genomic, transcriptomic, and proteomic investigations available in the PubMed literature database. Consequently, this information was used to identify relationship networks between the genes, their transcripts, their proteins and other lung cancer-relevant biological processes or pathways [30]–[32].

## Results

### Identification of Specific Common Tracts of Homozygosity (cTOH) in Individuals with Lung Cancer in the PLCO Cohort

**Figure 1:**
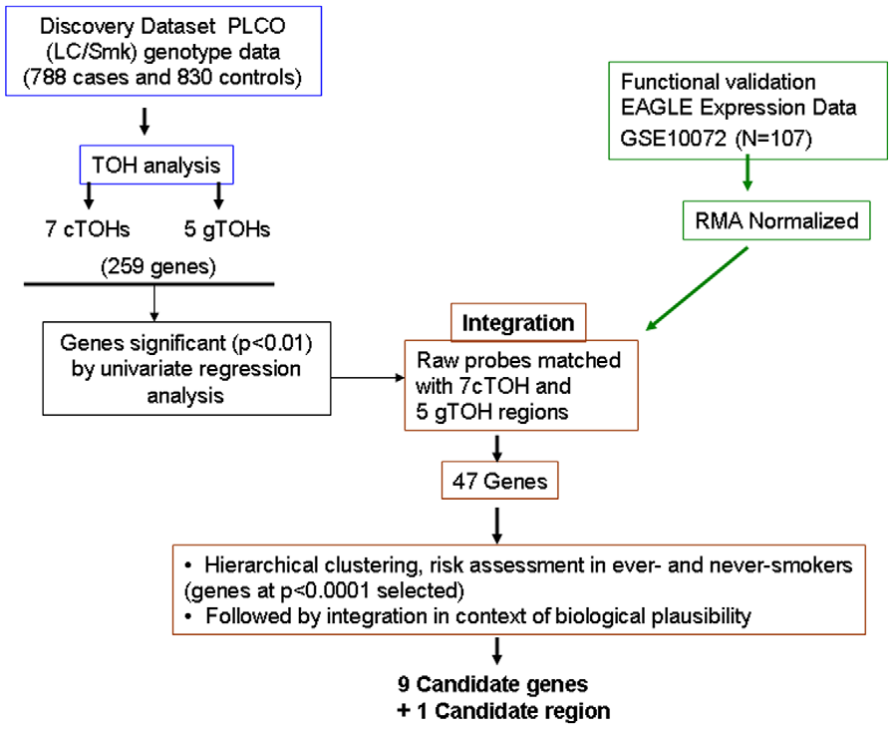
Study schema for the identification and functional genomic validation of significant cTOH and aTOH regions that are over- or under-represented in lung cancer cases. The schema represents the framework used to identify and subsequently integrate significant cTOHs and aTOHs (from the PLCO lung cancer screening trial) with global transcriptome datasets comparing lung cancers to normal lungs (from the EAGLE lung cancer screening trial). Multiple differentially expressed genes within the cTOHs and aTOHs had their candidacy prioritized initially based on statistical significance followed by biological plausibility (eg, relevant mouse models, reported to be somatically altered in sporadic lung cancers, relevant signaling pathways, etc) to finally obtain 9 “most plausible” candidate genes and one candidate genomic region. The latter is so designated because it was independently derived (by this current study) and subsequently found to overlap with the region previously identified in 3 previous studies as associated with lung cancer risk.

To address our central hypothesis that specific germline TOH is either over- or under-represented in lung-cancer cases over ancestry-matched controls, we initially screened for TOH regions in the PLCO-dataset (schema in Figure 1). We found a total of 91,460 TOHs across all samples with 44,725 TOHs in cases and 46,735 TOHs in controls. Average length of TOHs was 886 kb (median = 677.4 kb, 1^st^ quartile = 484.8 kb, 3^rd^ quartile = 956.3 kb) and average number of SNPs within each TOH 141.4 (median 121, 1^st^ quartile108, 3^rd^ quartile = 145). A total of 890 such cTOHs were identified across the genome, ranging in size 141.6–3421 kb (mean = 2144 kb, SD = 3115.6 kb, median = 1064 kb, 1^st^ quartile 623.9 kb, 3^rd^ quartile 2144 kb) and SNP-count of 100–413 (mean = 375, SD = 418, median = 215).

**Figure 2:**
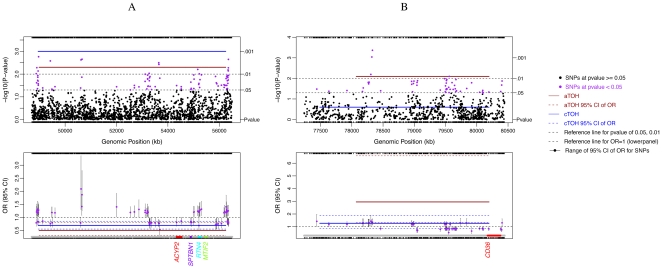
cTOH3/aTOH1 and aTOH4 regions under-represented in lung cancer cases compared to controls. Single-SNP association analysis was performed independently of TOH analysis and compared. The significant associations of single SNPs, and each TOH with lung cancer cases versus controls, and their respective 95% CI (colored dashed lines), are shown. Below each of the lower panels are candidate gene names (multi-colored) which were prioritized after testing for association between lung cancers and differential expression of each of the genes within and +/− 250 kb of the TOH, stratified by smoking status (p<0.0001; see Methods section). **A.** cTOH3/aTOH1 region (2 p16.3–16.1; brown line) significantly under-represented in lung cancer cases and GWAS-identified SNPs (purple dots) in the same region (top panel) with their respective corresponding risks as odds ratios (lower panel). **B.** aTOH4 (7q21.11; brown line) significantly under-represented in lung cancer cases and GWAS-identified SNPs (purple dots) in the region (top panel) with their corresponding lung-cancer risks as odds ratios (OR; lower panel).

**Figure 3:**
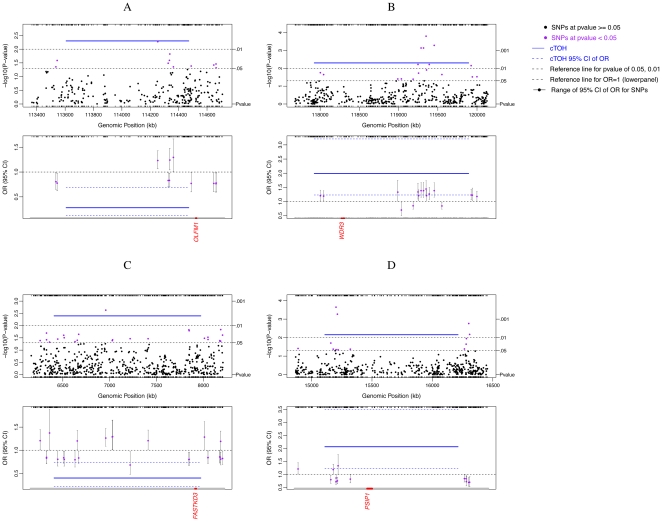
Lung cancer-associated cTOH1, cTOH2, cTOH5 and cTOH7 regions Single SNP association analysis was performed (independently of TOH analysis), after which the SNP association was compared to significant TOHs obtained with TOH analysis. The significant lung cancer-associated single SNPs, and TOH's namely cTOH1, cTOH2, cTOH5, and cTOH7, and their respective 95% CI are shown. The significant lung cancer association of aTOHs and SNPs in the region (top panel) and corresponding risk as odds ratios (lower panel) are shown in panels **A–D.** Below the lower panels are candidate genes which were prioritized after testing for association between lung cancer and differential expression of each of the genes within each significant TOH +/−250 kb TOH, stratified by smoking status (at p<0.0001; see Methods section).

By considering each cTOH as a genomic variant, we performed a case-control analysis adjusting for the effects of age, sex and smoking status. Seven cTOH regions were found to be significantly differentially represented between LC cases and controls based on p<0.01 (Table 1, Figure 2 A and Table S2) [38 cTOH regions were found at p<0.05 (data not shown)]. Three cTOH regions, cTOH2, 4 and 7 (within 1p12, 3p24.2–3p24.1 and 9p22.3, respectively), have odds ratios (OR) = 1.75–2.06 (p = 0.007–0.001), showing over-representation of these 3 cTOHs in lung-cancer cases over controls (Table 1 Table S2, and Figures 3C and 3D). The remaining four cTOH regions, cTOH1, 3, 5 and 6 (1p13.2, 2p16.3–2p16.1, 5p15.31 and 6p22.3–22.2) have OR = 0.28–0.69 (p = 0.006–0.001), showing that these cTOH's were under-represented in cases compared to controls (Table 1, Table S2, and Figures 3A and 3B).

**Table 1:** Covariate-adjusted significant (p<0.01) common tracts of homozygosity (cTOHs) over- or under-represented in lung cancer cases compared to controls.

cTOH region #	Chromosome region	Start rs #	End rs #	Length	# of SNPs	P-value^1^	FDR	q-value	OR^2^ (95%CI)
**1** *	1 p13.2	rs773610	rs4307568	864192	149	0.005	0.193	0.193	0.28 (0.12,0.69)
**2** *	1 p12	rs861153	rs10802112	1967456	313	0.005	0.193	0.193	1.99 (1.23,3.21)
**3** *	2 p16.3–16.1	rs733726	rs4672095	7302499	1789	0.001	0.076	0.076	0.69 (0.55,0.85)
**4**	3 p24.2–24.1	rs4858125	rs13066666	3081499	606	0.003	0.204	0.204	1.75 (1.21,2.52)
**5** *	5 p15.31	rs10040610	rs13173919	1565397	412	0.004	0.204	0.058	0.40 (0.22,0.74)
**6**	6 p22.3–22.2	rs6937402	rs7775425	1179898	310	0.006	0.312	0.215	0.51 (0.32,0.82)
**7**	9 p22.3	rs10511606	rs10962339	1101955	263	0.007	0.35	0.343	2.06 (1.22,3.49)

Pvalue^1^: p-value (obtained by a Wald test) of the effect of a cTOH region after adjusting for age, sex and smoking status in a logistic model.

OR^2^ (95%CI): Adjusted odds ratio (OR), with its 95% confidence interval, of the cTOH region associated with lung-cancer cases over controls.

**Figure 4:**
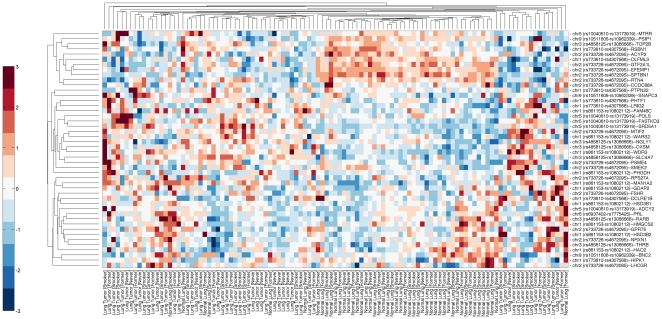
Clustering of gene expression profiles for genes residing within significant c/aTOH +/−250 kb regions. Bi-clustering of relative gene expression (horizontal) classified by “LC (tumor)+smoking status” and “normals+smoking status” (vertical). The acquisition, re-standardization and merging of expression array data with TOH regions are detailed in the Methods section. Red coloration on the heat map is relative over-expression of the genes, blue denotes relative under-expression and white no distinct relative expressional difference observed. The heat map represents the differential expression profiles of 47 genes that were selected after univariate analysis (see Methods section for details). The expression profiles of genes residing within and in proximity to the c/aTOH regions that are associated with tobacco use differentiate lung carcinomas from normal lung tissue.

*: regions containing genes differentially expressed in lung cancer (AC) versus normal lungs (see Figure 4).

rs #: SNP identification numbers.

Interestingly, interaction between smoking status and cTOH3 (rs733726∼ rs4672095 [2p16.3–2p16.1]; Table 1) was observed (p<0.03, Table S3). While age-, sex- and smoking-status-adjusted OR for cTOH 3 is 0.69 (Table 1, Figure 2 A), cTOH3 is 2-fold (OR = 1.8) over-represented in non-smoking cases over non-smoking controls, whereas cTOH3 is significantly under-represented in ever-smoking cases over ever-smoking controls [OR 0.78 (previous smokers) and 0.34 (current smokers), respectively, p = 0.009–0.026] (Table S3 B).

### Identification of Allelically-Matching Groups (aTOH) within cTOHs in Lung-Cancer Cases and Controls

The aTOHs may provide genetic background or ancestry-related information, hence a biological meaningful association with the lung-cancer phenotype. The number of aTOHs in each cTOH ranges from 1 to 111. We conducted an independent (of cTOHs identified) case-control analysis followed by adjusting for the effects of age, sex and smoking status on the lung-cancer phenotype. In this manner, we identified 5 aTOHs (within 2p16.3–2p16.1, 3p25.3, 5q11.2–12.1, 7q21.11 and 13q31.1–31.3) that are significantly differentially represented between cases and controls (based on p<0.01; Table 2). Notably, only aTOH1 with OR of 0.5 (Table 2), was derived from parent cTOH3 (2p16.3–16.1) where both cTOH3 and aTOH1 are significantly under-represented in lung-cancer cases compared to controls (OR = 0.69 and 0.5, p = 0.001 and 0.005, respectively; Figure 2, Tables 1 and 2). The remaining aTOH regions, aTOH2, 3, 4 and 5 (within 3p25.3, 5q11.2–12.1, 7q21.11 and 13q31.1–31.3, respectively) have OR = 3.97, 0.23, 2.95 and 0.39, respectively (p = 0.001–0.008; Table 2).

**Table 2:** Covariate-adjusted significant (p<0.01) allelically-matched tracts of homozygosity (aTOHs) over- or under-represented in lung cancer cases compared to controls.

Chromosome region	cTOH & its aTOH	Start rs #	End rs #	Length	# of SNPs	N(%) Cases	N(%) Controls	P-value^1^	FDR	q-value	OR^2^ (95% CI)
2p16.3–16.1*	cTOH region	rs733726	rs4672095	7302499	1789	31(3.9))	55(6.6)	0.001	0.076	0.076	0.69 (0.55,0.85)
2p16.3–16.1*	aTOH1	rs2215911	rs4672095	7264821	1774	31(3.9))	55(6.6)	0.005	0.600	0.600	0.50 (0.31,0.81)
3p25.3	cTOH region	rs6804473	rs9990174	980014	316	28(3.6)	8(1.0)	0.055	0.737	0.737	1.65 (0.99,2.74)
3p25.3	aTOH2	rs6804473	rs9990174	980014	316	28(3.6)	8(1.0)	0.001	0.091	0.091	3.97 (1.75,8.99)
5q11.2–12.1*	cTOH region	rs1363793	rs410850	4097344	835	5(0.6)	22(2.7)	0.021	0.391	0.111	0.73 (0.55,0.95)
5q11.2–12.1*	aTOH3	rs1363793	rs379212	4067496	820	5(0.6)	22(2.7)	0.004	0.213	0.207	0.23 (0.08,0.63)
7q21.11*	cTOH region	rs2018955	rs16886849	2708287	696	25(3.2)	9(1.1)	0.250	0.731	0.731	1.27 (0.85,1.89)
7q21.11*	aTOH4	rs38094	rs16886849	2124458	413	25(3.2)	9(1.1)	0.008	0.680	0.594	2.95 (1.33,6.58)
13q31.1–31.3*	cTOH region	rs1361540	rs9524302	15043999	2438	14(1.8)	31(3.7)	0.377	0.643	0.628	0.91 (0.73,1.13)
13q31.1–31.3*	aTOH5	rs370787	rs9584099	13011025	2046	14(1.8)	31(3.7)	0.006	0.342	0.244	0.39 (0.20,0.76)

Pvalue^1^: p-value obtained by Wald test of the effect of the aTOH region after adjusting for age, sex and smoking status in a logistic model.

OR^2^ (95%CI): Adjusted odds ratio (OR) with its 95% confidence interval of the aTOH region.

*: regions containing genes differentially expressed in lung carcinomas (mainly AC) versus normal lung (see Figure 3) derived from experimentally validated expression arrays.

### Functional Genomic Validation by Integration of Significant cTOH and aTOH Regions with Global Transcriptome Datasets

We next turned our attention to look for biologically plausible genes, i.e., one or a subset of all genes, located within and in proximity (+/−250 kb) to significant c/aTOH's and that may be germane to lung cancer risk. To fine map the TOHs containing lung-cancer-related genes and to functionally validate our genomic data, we integrated our significant TOH regions with gene expression data derived from lung cancer patients in the EAGLE study [27] (Figure 1). This dataset was derived from a population of European ancestry (selection criteria described in the Methods section) and also served as our functional validation series. We were able to filter out genes within the significant c/aTOH regions to 46 genes based on differential expression in univariate analysis alone (Figures 1 and 4). With further risk analyses and integration with known organ-specific function and signaling pathway roles, we ended up with a final shortlist of 9 most-plausible lung cancer-risk genes and one candidate genomic region (p<0.0001; Table 3 and Figure 1; see Discussion).

**Table 3:** Risk associated with differential expression of genes within aTOHs and cTOHs stratified by smoking status.

		Ever smokers		Never smokers	
cTOH/aTOH (Chromosome region)	Genes	Odds ratio (95% CI)	p-values	Odds ratio (95% CI)	p-values
cTOH1 (1p13.2)	*OLFML3*	0.03 (0.01, 0.12)	<.0001	0.11 (0.02, 0.59)	0.0064
cTOH2 (1p12)	*WDR3*	21.27 (6.39, 70.73)	<.0001	97.5 (7.9, 1203.06)	<.0001
cTOH3/aTOH1 (2 p16.3–16.1)	*ACYP2*	0.08 (0.03, 0.24)	<.0001	0.39 (0.09, 1.67)	NS
cTOH3/aTOH1 (2 p16.3–16.1)	*MTIF2*	18.75(5.43, 64.77)	<.0001	8.25 (1.65, 41.25)	0.0071
cTOH3/aTOH1 (2 p16.3–16.1)	*RTN4*	0.1 (0.03, 0.3)	<.0001	0.02 (0, 0.24)	0.0001
cTOH3/aTOH1 (2 p16.3–16.1)	*SPTBN1*	0.00 (0, 0.03)	<.0001	0.00 (0, 0.08)	<.0001
cTOH5 (5p15.31)	*FASTKD3*	11.6 (3.69, 36.47)	<.0001	3.54 (0.78, 16.03)	0.0954
cTOH7 (9p22.3)	*PSIP1*	0.06 (0.02, 0.2)	<.0001	0.005(0.0003, 0.084)	<.0001
aTOH4 (7q21.11)	*CD36*	0 (0, 0.02)	<.0001	0.00 (0.00, ∞)	NS

P-value1: p-value obtained by Chi squared test, for each gene within each cTOH/aTOH stratified by smoking status.

OR^2^ (95%CI): Adjusted odds ratio (OR) with its 95% confidence interval of the aTOH region in cases over controls.

∞ infinity.

NS: Not significant (p>0.1).

We particularly examined association of the TOHs harboring these 9 genes and smoking status. Relatedly, the 9 differentially expressed genes within the 6 cTOH/aTOH are germane in ever-smokers compared to 3 that are germane in both ever- and never-smokers [(p<0.0001), Table 3]. One important exception is *SBTBN1* and *RTN4* within cTOH3/aTOH1 (2p16.3–16.1), where over-expression occurs almost exclusively in controls relative to lung-cancer cases, irrespective of smoking status (OR = 0.000 and 0.08, p<0.0001; Table 3, Figures 2A and 4). *ACYP2* (OR = 0.08, p<0.0001), also within this TOH, is under-expressed in ever-smokers associated with decreased lung-cancer-risk, but its differential expression is not germane in never-smokers (Table 3, Figures 2A and 4). Overall, unique differential expression signatures were observed for gene groups within a/cTOHs as shown in Table 3 and Figure 4. Analysis of expression profiles of genes in other aTOHs, eg, *CD36* in aTOH4 (7q21.11), showed under-expression in cases amongst ever-smokers (p<0.0001; Table 3 and Figure 2B).

Expression profiles of the genes located in other significant cTOHs, cTOH1, 2, 5 and 7 (1 p13.2, 1p12, 5p15.31 and 9p22.3, respectively; Table 1) were analyzed. *OLFML3* (1p12; Figure 3A), was under-expressed in ever-smoking cases compared to never-smoking cases consistent with a reduced risk as portrayed by OR's<1 (Table 3 and Figure 4). In contrast *WDR3* (on 1p12; Figure 3B) showed significant relative over-expression irrespective of the smoking status, consistent with the TOH-relevant OR>1 (Table 3 and Figure 4). *FASTKD3* (on 5p15.31; Figure 3C) showed significant relative over-expression in ever-smoking lung-cancer cases compared to never-smoking cases, consistent with the TOH-relevant OR>1 (Table 3 and Figure 4). *PSIP1* (on 9p22.3; Figure 3D) was significantly under-expressed in both ever- and never-smoking cases, OR<1 (Table 3 and Figure 4). In general, we observed unique and similar expressional signatures for specific gene-sets (Table 3, Figure 4). For example, we observed net under-expression of a gene set within the cTOH3 in lung cancer cases who are smokers (OR<1) [Table 3, Figure 4].

## Discussion

Identifying risk factors, whether genetic or environmental, for malignancies, including lung cancer, is a start for early diagnosis, and tailoring heightened surveillance and prevention. The common variant-common cancer hypothesis prevalent in the last decade led to GWAS yielding common SNPs within 15q25, 5p15, and 6p21 associated with lung cancer [5]–[10], accounting for ∼3% of all lung cancers. Based on the working hypothesis that other genomic factors predisposing to or lowering lung cancer risk must exist, we performed a genome-wide case-control analysis for long TOHs, each of which harbors one to several lung-cancer-predisposing or protective loci (most likely of low to moderate penetrance). We identified 7 cTOHs and 5 aTOHs that are significantly over- or under-represented in lung cancer cases versus controls, after adjusting for age, gender and smoking status. Interestingly, we found specific cTOH/aTOHs associated with cases over controls independent of these covariates, with others dependent on smoking status.

Importantly, our identified significant cTOH and aTOH regions have been functionally validated by integrating differential expression of specific genes residing in these critical intervals, previously shown to play at least a somatic role in sporadic human lung carcinomas, in murine models and/or participate in neoplasia-associated signaling pathways (Table S4). We believe that agnostically searching for cTOH and aTOH and then integrating with expression data are powerful methods for finding, and at the same time functionally genomically validating, new lung cancer-risk regions and genes. Three previous lung cancer GWAS studies have identified the 5p15 region to be associated with lung cancer cases [4]–[10]. cTOH5 lies within 5p15.31 (our “candidate genomic region” after integrative analysis) and is 11-fold over-represented in ever-smoking lung cancer cases and 3-fold in never-smoking lung cancer cases. This serves as a strong positive control. We have also identified a new candidate gene *FASTKD3*, beyond those previously postulated, by integration of expression with significant TOH in this region (Table S4).

We found only one TOH region where a significant aTOH lies within its parent cTOH: aTOH 1 (2p16.3–16.1) and its parent cTOH3, whose presence appears to confer a protective effect against lung cancer in ever-smoking cases (OR<0.7, ie, over-represented in controls versus cases; Tables 1 and 2). Differential expression of a group of genes in this region seems to be equally protective against lung cancer irrespective of smoking status or history (Table 3, Figure 4). Eg, *SPTBN1* codes for a beta-spectrim which plays a role in decreasing cell surface recruitment of CD45 and CD3, and abrogating T-cell function [33]. Accordingly, increased *SPTBN1* expression (and over-representation of aTOH1/cTOH3 in controls over cases) could plausibly protect against lung cancer by increasing immune surveillance, given that we know that smoking suppresses the CD4/CD8 T cell ratio [34]. While there is plausible existing evidence that under-expression of genes within cTOH3/aTOH1 (Table 3, Table S4, Figure 4) would be protective through various mechanisms [35], we do not know what undiscovered mechanisms result in further mitigation of smoking-associated lung-cancer risk. Unlike the other genes in aTOH1/cTOH3, *MTIF2* over-expression is associated with its TOH differentially associated with cases and controls. MTIF2 is a mitochondrial translation-initiation factor that partners with RNaseL. *In vitro* over-expression of MTIF2 stabilizes mitochondrial RNA, inhibits apoptosis induced by interferon-alpha and partially reverses alpha-interferon-cell growth inhibition [36]. Thus, this mechanism lends plausibility to the high OR in lung cancer cases over controls and in ever-smoking cases over never-smoking cases (OR = 18.75 vs 8.25; Table 3, Table S4, Figure 4). Amongst the other “most plausible” risk genes worthy of mention are *CD36* (aTOH4) and *PSIP1* (cTOH7), both of which have plausible roles in lung function development, and genetic alterations (especially in the oncogene *PSIP1*) may well lead to NSCLC development (detailed in Table S4). Major vault protein (MVP), implicated in the regulation of cellular signaling cascades and multidrug resistance, has been shown to interact with IFNgamma-regulated gene (CD36) in the H65 lung cancer cell model [37]. Hence CD36, may be important in the development of lung cancer. *OLFM* (cTOH1) and *WDR3* (cTOH2) have been implicated in apoptosis and cell cycle regulation in cancer cells (Table S4). The two genes may be important in the regulation of lung carcinoma cell proliferation.

In this study, differences in expression profiles displayed by genes within significant TOH regions can be interpreted as due to intra-c/aTOH or inter-c/aTOH composition and cross-talk, together with environmental influences (Table 3, Figures 2 and 3). These genes likely work in conjunction with each other to either magnify or suppress the risk of lung cancer, more so in the presence or absence of tobacco (Tables 1, 2 and 3). A gene set effect (intra-gene-gene signature) within a TOH is most likely controlled by the direction of the OR and whether the genes are over- or under-expressed. Eg, cTOH3 with OR<0.7 will most likely have a gene-set that in combination relays a net protective effect in lung cancer cases who are exposed to tobacco (Tables 1, 2 and 3).

Systematically integrating differential expression of specific genes residing in critical intervals such as tracts of homozygosity, have revealed new candidate lung cancer-risk-genes, as well as genes previously shown to play a somatic role in sporadic human lung carcinomas, in murine models and/or participate in neoplasia-associated signaling pathways. This regional approach of systems integration with identification of regional subsets of genes can complement classical analyses which only consider single genes represented by GWAS-associated SNP-risk.

## Supporting Information

Table S1 **Effects of age, gender and smoking status on lung cancer risk.** The table shows the effects of age, gender and smoking status on lung cancer in a PLCO cohort. A logistic regression model was used to obtain an adjusted odds ratio with a 95% confidence interval.
(DOC)

Table S2 **Covariate-adjusted significant (p<0.01) association of specific common tracts of homozygosity (cTOHs) with lung cancer.** This table shows the effect of a cTOH region on the risk of lung cancer after adjusting for age, sex and smoking status in a logistic model. The adjusted odds ratio (OR), with its 95% confidence interval, of the cTOH region associated with lung-cancer are also shown.
(DOC)

Table S3 A. Interaction coefficient estimates of cTOH3 and age, and of cTOH3 and smoking status on lung cancer. The result in this table is from an interaction analysis between smoking status and cTOH3. The estimated standard error and coefficient estimate of the logistic model the p-value (significant at p<0.05) are shown. **B. Risk associated with interaction between cTOH3 and smoking status on lung cancer.** This table shows the risk associated with interaction between smoking status and cTOH3. The odds ratio was calculated using the reference as male, age < = 59, nonsmoking and absence of cTOH 3.
(DOC)

Table S4 **Summary of candidate genes putatively involved in lung cancer predisposition.** This a summary of the final candidate gene list within specific significant TOH (i.e. cTOH or aTOH) regions. The two columns on the right of the candidate genes show previous experiments and animal models. This validates the putative roles of the selected candidate genes in lung cancer.
(DOC)

## References

[1] Turnbull C, Hodgson S (2005). Genetic predisposition to cancer.. Clin Med.

[2] Frank SA (2004). Genetic predisposition to cancer - insights from population genetics.. Nat Rev Genet.

[3] Mincey BA (2003). Genetics and the management of women at high risk for breast cancer.. Oncologist.

[4] Landi MT, Chatterjee N, Yu K, Goldin LR, Goldstein AM (2009). A genome-wide association study of lung cancer identifies a region of chromosome 5p15 associated with risk for adenocarcinoma.. Am J Hum Genet.

[5] Huang RT (1978). Cell adhesion mediated by glycolipids.. Nature.

[6] Amos CI, Wu X, Broderick P, Gorlov IP, Gu J (2008). Genome-wide association scan of tag SNPs identifies a susceptibility locus for lung cancer at 15q25.1.. Nat Genet.

[7] Thorgeirsson TE, Geller F, Sulem P, Rafnar T, Wiste A (2008). A variant associated with nicotine dependence, lung cancer and peripheral arterial disease.. Nature.

[8] McKay JD, Hung RJ, Gaborieau V, Boffetta P, Chabrier A (2008). Lung cancer susceptibility locus at 5p15.33.. Nat Genet.

[9] Wang Y, Broderick P, Webb E, Wu X, Vijayakrishnan J (2008). Common 5p15.33 and 6p21.33 variants influence lung cancer risk.. Nat Genet.

[10] Rafnar T, Sulem P, Stacey SN, Geller F, Gudmundsson J (2009). Sequence variants at the TERT-CLPTM1L locus associate with many cancer types.. Nat Genet.

[11] Gao Y, Goldstein AM, Consonni D, Pesatori AC, Wacholder S (2009). Family history of cancer and nonmalignant lung diseases as risk factors for lung cancer.. Int J Cancer.

[12] Li X, Hemminki K (2004). Inherited predisposition to early onset lung cancer according to histological type.. Int J Cancer.

[13] Henry I, Bonaiti-Pellie C, Chehensse V, Beldjord C, Schwartz C (1991). Uniparental paternal disomy in a genetic cancer-predisposing syndrome.. Nature.

[14] Rudan I, Rudan D, Campbell H, Carothers A, Wright A (2003). Inbreeding and risk of late onset complex disease.. J Med Genet.

[15] Shami SA, Qaisar R, Bittles AH (1991). Consanguinity and adult morbidity in Pakistan.. Lancet.

[16] Rudan I (1999). Inbreeding and cancer incidence in human isolates.. Hum Biol.

[17] Simpson JL, Martin AO, Elias S, Sarto GE, Dunn JK (1981). Cancers of the breast and female genital system: search for recessive genetic factors through analysis of human isolate.. Am J Obstet Gynecol.

[18] Bhattacharya P, Duttagupta C, Sengupta S (2002). Proline homozygosity in codon 72 of p53: a risk genotype for human papillomavirus related cervical cancer in Indian women.. Cancer Lett.

[19] Assie G, LaFramboise T, Platzer P, Eng C (2008). High frequency of germline genomic homozygosity associated with cancer cases.. JAMA.

[20] Bacolod MD, Schemmann GS, Wang S, Shattock R, Giardina SF (2008). The signatures of autozygosity among patients with colorectal cancer.. Cancer Res.

[21] Prorok PC, Andriole GL, Bresalier RS, Buys SS, Chia D (2000). Design of the Prostate, Lung, Colorectal and Ovarian (PLCO) Cancer Screening Trial.. Control Clin Trials.

[22] Patterson N, Price AL, Reich D (2006). Population structure and eigenanalysis.. PLoS Genet.

[23] Lao O, Lu TT, Nothnagel M, Junge O, Freitag-Wolf S (2008). Correlation between genetic and geographic structure in Europe.. Curr Biol.

[24] Lencz T, Lambert C, DeRosse P, Burdick KE, Morgan TV (2007). Runs of homozygosity reveal highly penetrant recessive loci in schizophrenia.. Proc Natl Acad Sci U S A.

[25] Storey JD, Tibshirani R (2003). Statistical significance for genomewide studies.. Proc Natl Acad Sci U S A.

[26] Purcell S, Neale B, Todd-Brown K, Thomas L, Ferreira MA (2007). PLINK: a tool set for whole-genome association and population-based linkage analyses.. Am J Hum Genet.

[27] Landi MT, Dracheva T, Rotunno M, Figueroa JD, Liu H (2008). Gene expression signature of cigarette smoking and its role in lung adenocarcinoma development and survival.. PLoS One.

[28] Hochreiter S, Clevert DA, Obermayer K (2006). A new summarization method for Affymetrix probe level data.. Bioinformatics.

[29] Eisen MB, Spellman PT, Brown PO, Botstein D (1998). Cluster analysis and display of genome-wide expression patterns.. Proc Natl Acad Sci U S A.

[30] Chen H, Sharp BM (2004). Content-rich biological network constructed by mining PubMed abstracts.. BMC Bioinformatics.

[31] Jupiter D, Chen H, VanBuren V (2009). STARNET 2: a web-based tool for accelerating discovery of gene regulatory networks using microarray co-expression data.. BMC Bioinformatics.

[32] Dennis G, Sherman BT, Hosack DA, Yang J, Gao W (2003). DAVID: Database for Annotation, Visualization, and Integrated Discovery.. Genome Biol.

[33] Pradhan D, Morrow J (2002). The spectrin-ankyrin skeleton controls CD45 surface display and interleukin-2 production.. Immunity.

[34] Fusby JS, Kassmeier MD, Palmer VL, Perry GA, Anderson DK (2010). Cigarette smoke-induced effects on bone marrow B-cell subsets and CD4+:CD8+ T-cell ratios are reversed by smoking cessation: influence of bone mass on immune cell response to and recovery from smoke exposure.. Inhal Toxicol.

[35] Yue W, Dacic S, Sun Q, Landreneau R, Guo M (2007). Frequent inactivation of RAMP2, EFEMP1 and Dutt1 in lung cancer by promoter hypermethylation.. Clin Cancer Res.

[36] Le Roy F, Silhol M, Salehzada T, Bisbal C (2007). Regulation of mitochondrial mRNA stability by RNase L is translation-dependent and controls IFNalpha-induced apoptosis.. Cell Death Differ.

[37] Steiner E, Holzmann K, Pirker C, Elbling L, Micksche M (2006). The major vault protein is responsive to and interferes with interferon-gamma-mediated STAT1 signals.. J Cell Sci.

