# Supplementary material for: Integrative Genomic Analysis Reveals Extended Germline Homozygosity with Lung Cancer Risk in the PLCO Cohort
Source: PLoS One. 2012 Feb 27;7(2):e31975. doi: 10.1371/journal.pone.0031975 (PMC3288062; doi:10.1371/journal.pone.0031975)
Supplement: Table S1 — Effects of age, gender and smoking status on lung cancer risk. The table shows the effects of age, gender and smoking status on lung cancer in a PLCO cohort. A logistic regression model was used to obtain an adjusted odds ratio with a 95% confidence interval. (DOC) [file pone.0031975.s001.doc]

**Table S1. Effects of age, gender and smoking status on lung cancer risk**

|  |  | **Cases (%) (n=788)** | **Controls (%) (n=830)** | **P-value^1^** | **OR (95%CI)^2^** |
| --- | --- | --- | --- | --- | --- |
| **Gender** | Male | 490(50.7%) | 477(49.3%) | 0.267 |  |
|  | Female | 298(45.8%) | 353(54.2%) |  | 0.87 (0.71,1.08) |
| **Smoking Status** | Never | 81 (51.9%) | 75 (48.1%) | 2.00E-11 |  |
|  | Previous | 429 (61.0%) | 274 (39.0%) |  | 1.48 (1.04,2.13) |
|  | Current | 278 (36.6%) | 481 (63.4%) |  | 0.62 (0.43,0.88) |
| **Age** | 0 (<=59) | 120 (40.4%) | 177 (59.6%) |  |  |
|  | 1 (60~64) | 184 (41.3%) | 262 (58.7%) |  |  |
|  | 2 (65~69) | 242 (47.5%) | 267 (52.5%) |  |  |
|  | 3 (70~74) | 173 (58.2%) | 124 (41.8%) |  |  |
|  | 4 (75~79) | 53 (100%) | 0 (100%) |  |  |
|  | 5 (80~84) | 16 (100%) | 0 (100%) |  |  |
|  | mean | 1.41 | 1.88 | 2.00E-09 | 1.38 (1.26,1.51) |

P-value^1^: P-value from testing the effect of the covariate on the disease by applying a logistic model after adjusting for the other two covariates.

OR(95%CI)^2^: Adjusted odds ratio (95% confidence interval) of the covariate on the disease by applying a logistic model after adjusting for the other two covariates
