# Supplementary material for: Integrative Genomic Analysis Reveals Extended Germline Homozygosity with Lung Cancer Risk in the PLCO Cohort
Source: PLoS One. 2012 Feb 27;7(2):e31975. doi: 10.1371/journal.pone.0031975 (PMC3288062; doi:10.1371/journal.pone.0031975)
Supplement: Table S2 — Covariate-adjusted significant (p<0.01) association of specific common tracts of homozygosity (cTOHs) with lung cancer. This table shows the effect of a cTOH region on the risk of lung cancer after adjusting for age, sex and smoking status in a logistic model. The adjusted odds ratio (OR), with its 95% confidence interval, of the cTOH region associated with lung-cancer are also shown. (DOC) [file pone.0031975.s002.doc]

**Table S2. Covariate-adjusted significant (p<0.01) association of specific common tracts of homozygosity (cTOHs) with lung cancer**

| **cTOH region#** | **Chr region^rs^** | **# of SNPs** | **N Cases** | **N control** | **N female** | **N Male** | **N Non Smk** | **N Pre Smk** | **N Curr Smk** | **Mean Age Cat cTOH** | **Mean Age Cat ncTOH** | **P- value^1^** | **FDR** | **q-value** | **OR^2^ (95%CI)** |
| --- | --- | --- | --- | --- | --- | --- | --- | --- | --- | --- | --- | --- | --- | --- | --- |
| 1^ | 1 p13.2 | 149 | 7 | 22 | 11 | 18 | 5 | 13 | 11 | 1.66 | 1.64 | 0.005 | 0.193 | 0.193 | 0.28 (0.12,0.69) |
| 2^ | 1 p12 | 313 | 53 | 30 | 38 | 45 | 12 | 39 | 32 | 1.57 | 1.64 | 0.005 | 0.193 | 0.193 | 1.99 (1.23,3.21) |
| 3^ | 2 p16.3-16.1 | 1789 | 252 | 326 | 227 | 351 | 49 | 266 | 263 | 1.63 | 1.64 | 0.001 | 0.076 | 0.076 | 0.69 (0.55,0.85) |
| 4 | 3 p24.2-24.1 | 606 | 86 | 57 | 51 | 92 | 16 | 64 | 63 | 1.55 | 1.64 | 0.003 | 0.204 | 0.204 | 1.75 (1.21,2.52) |
| 5^ | 5 p15.31 | 412 | 16 | 38 | 19 | 35 | 3 | 19 | 32 | 1.87 | 1.63 | 0.004 | 0.204 | 0.058 | 0.40 (0.22,0.74) |
| 6 | 6 p22.3-22.2 | 310 | 29 | 55 | 33 | 51 | 6 | 39 | 39 | 1.6 | 1.64 | 0.006 | 0.312 | 0.215 | 0.51 (0.32,0.82) |
| 7^ | 9 p22.3 | 263 | 46 | 24 | 34 | 36 | 11 | 34 | 25 | 1.66 | 1.64 | 0.007 | 0.35 | 0.343 | 2.06 (1.22,3.49) |

P-value^1^: p-value (obtained by a Wald test) of the effect of a cTOH region after adjusting for age, sex and smoking status in a logistic model

OR^2^ (95%CI): Adjusted odds ratio (OR), with its 95% confidence interval, of the cTOH region associated with lung-cancer cases over controls

^: regions containing genes differentially expressed in lung cancer (AC) versus normal lungs (see Figure 4)

**^rs^**: SNPs defining the regions are shown in Table 1

Chr: Chromosome

N: number of individuals

nc: individuals with absence of cTOH, denoted as ncTOH.

Non Smk: non smokers (=never smokers)

Pre Smk: previous smokers

Curr Smk: current smokers
