# Supplementary material for: Integrative Genomic Analysis Reveals Extended Germline Homozygosity with Lung Cancer Risk in the PLCO Cohort
Source: PLoS One. 2012 Feb 27;7(2):e31975. doi: 10.1371/journal.pone.0031975 (PMC3288062; doi:10.1371/journal.pone.0031975)
Supplement: Table S3 — A. Interaction coefficient estimates of cTOH3 and age, and of cTOH3 and smoking status on lung cancer. The result in this table is from an interaction analysis between smoking status and cTOH3. The estimated standard error and coefficient estimate of the logistic model the p-value (significant at p<0.05) are shown. B. Risk associated with interaction between cTOH3 and smoking status on lung cancer. This table shows the risk associated with interaction between smoking status and cTOH3. The odds ratio was calculated using the reference as male, age < = 59, nonsmoking and absence of cTOH 3. (DOC) [file pone.0031975.s003.doc]

**Table S3**

**A. Interaction coefficient estimates of cTOH3 and age, and of cTOH3 and smoking status on lung cancer**

| **Variable** | **Estimate (Std.Error)^1^** | **exp^(Estimate)^** | **Pvalue^2^** |
| --- | --- | --- | --- |
| **Gender (Female)** | -0.14(0.11) | 0.87 | 0.183 |
| **Age** | 0.35(0.06) | 1.42 | <0.001* |
| **Previous Smoking** | 0.76(0.22) | 2.14 | 0.001 |
| **Current Smoking** | -0.20(0.22) | 0.82 | 0.37 |
| **cTOH3 (presence)** | 0.59(0.40) | 1.80 | 0.146 |
| **Age by cTOH3 (presence)** | -0.07(0.10) | 0.93 | 0.506 |
| **Previous Smoking by cTOH3 (presence)** | -1.01(0.39) | 0.36 | 0.009* |
| **Current Smoking by cTOH3 (presence)** | -0.87(0.39) | 0.42 | 0.026* |

Estimate (Std.Error)^1^: the coefficient estimate (and standard error) of the logistic model

Pvalue^2^: p-value of Wald-test in the logistic model

*: significant at p<0.05

**B. Risk associated with interaction between cTOH3 and smoking status on lung cancer**

|  | **Estimate** | **OR^3^** |
| --- | --- | --- |
| **Gender (Female)** | -0.14 | 0.87 |
| **Age** | 0.35 | 1.42 |
| **cTOH 3 presence in Non smokers** | 0.59 | 1.80 |
| **cTOH 3 presence in Previous smokers** | -0.25 | 0.78 |

OR^3^: the odds ratio with baseline is male, age <=59, nonsmoking and absence of cTOH 3
