# Supplementary material for: Integrative Genomic Analysis Reveals Extended Germline Homozygosity with Lung Cancer Risk in the PLCO Cohort
Source: PLoS One. 2012 Feb 27;7(2):e31975. doi: 10.1371/journal.pone.0031975 (PMC3288062; doi:10.1371/journal.pone.0031975)
Supplement: Table S4 — Summary of candidate genes putatively involved in lung cancer predisposition. This a summary of the final candidate gene list within specific significant TOH (i.e. cTOH or aTOH) regions. The two columns on the right of the candidate genes show previous experiments and animal models. This validates the putative roles of the selected candidate genes in lung cancer. (DOC) [file pone.0031975.s004.doc]

# Supporting Supplementary Data for Integrative genomic analysis reveals extended germline homozygosity and lung cancer risk in the PLCO cohort

Mohammed S Orloff^1,3^  Li Zhang^1,2^, Gurkan Bebek^1,6^, and Charis Eng^1,3,4,5,6*^

**Supplementary Tables**

**Table S4. Summary of candidate genes putatively involved in lung cancer predisposition**

| **cTOH/aTOH** | **Genes, Expression and smoking status** | **Experiments** | **Published supporting evidence** |
| --- | --- | --- | --- |
| **cTOH3/aTOH1** | *ACYP2* † | *Somatic* | An acylphosphatase, controls the Na+/K+-ATPase and its over-expression has been shown to increase metastases in colorectal cancers [1] and to be part of a proteomic profile that portends high risk of death from NSCLC, even stage I presentations [2] |
|  | *SBTBN1* |  | Codes for SPTBN1, a beta-spectrim which plays a role in decreasing cell surface recruitment of CD45 and CD3, and abrogating T-cell function[3] |
|  | *MTIF2* **­** |  | *In vitro* over-expression of MTIF2 stabilizes mitochondrial RNA, inhibits apoptosis induced by interferon-alpha and partially reverses alpha-interferon-cell growth inhibition [4] |
|  | *RTN4,* † | *cell line* | Previously reported to be markedly down-regulated SCC, but was expressed in normal lung tissues [5] |
| **aTOH4** | *CD36* †, |  | Previous reports has shown that a decreased expression of CD36 in adenocarcinoma when compared with small cell lung cancer (SCLC) [6]. This analysis was done using 2 published microarray data (GSE7670 and GSE10072). |
| **cTOH5** | *FASTKD3* |  | An array comparative genomic hybridization approach, has also shown that FASTKD3 is one of nine genes within 5p15.33-31 that is associated with SCLC tumors [7] |
| **cTOH2** | *OLFML3* |  | LIke WDR3. OLFML3 is linked to apoptosis [8] |
|  | *WDR3* | *Cell line* | WDR3 has been shown to have an essential function in 40S ribosomal subunit synthesis and in ribosomal stress signaling to p53-mediated regulation of cell cycle progression in cancer cells [9] |
| **cTOH7** | *PSIP1* | *Rat and cell line* | LEDGF [PSIP1] is an oncogenic protein that controls a caspase independent lysosomal cell death pathway [10] . Ectopic expression of LEDGF [PSIP1] p75 in C6 rat glioma and in H1299 human non small cell lung carcinoma induced VEGF C expression in vitro [11] |

†: genes validated by current study

**Supplementary References**

1. Riley HD, Macnab J, Farrell TJ, Cohn K (1997) The expression of acylphosphatase is associated with the metastatic phenotype in human colorectal tumors. Carcinogenesis18: 2453-5.

2. Yanagisawa K, Tomida S, Shimada Y, Yatabe Y, Mitsudomi T, et al. (2007) T. A 25-signal proteomic signature and outcome for patients with resected non-small-cell lung cancer. J Natl Cancer Inst 99: 858-67.

3. Pradhan D, Morrow J. (2002) The spectrin-ankyrin skeleton controls CD45 surface display and interleukin-2 production Immunity 17: 303-15.

4. Le Roy F, Silhol M, Salehzada T, Bisbal C (2007) Regulation of mitochondrial mRNA stability by RNase L is translation-dependent and controls IFNalpha-induced apoptosis Cell Death Differ 14:1406-13.

5. Shimakage M, Inoue N, Ohshima K, Kawahara K, Oka T, et al. (2006) Down-regulation of ASY/Nogo transcription associated with progression of adult T-cell leukemia/lymphoma. Int J Cancer 119:1648-53.

6. Wang G, Ye Y, Zheng W, Ma W. (2010) Identification of candidate genes for lung adenocarcinoma using Toppgene. Zhongguo Fei Ai Za Zhi 13: 282-6.

7. Voortman J, Lee JH, Killian JK, Suuriniemi M, Wang Y, Lucchi M, et al. (2010) Array comparative genomic hybridization-based characterization of genetic alterations in pulmonary neuroendocrine tumors. Proc Natl Acad Sci U S A 107: 13040-5.

8. Bergstrom U, Olsson JA, Hvidsten TR, Komorowski J, Brandt I (2007) Differential gene expression in the olfactory bulb following exposure to the olfactory toxicant 2,6-dichlorophenyl methylsulphone and its 2,5-dichlorinated isomer in mice. Neurotoxicology 28: 1120-1128.

9. McMahon M, Ayllon V, Panov KI, O'Connor R. (2010) Ribosomal 18 S RNA processing by the IGF-I-responsive WDR3 protein is integrated with p53 function in cancer cell proliferation. J Biol Chem 285: 18309-18318.

10. Daugaard M, Kirkegaard-Sorensen T, Ostenfeld MS, Aaboe M, Hoyer-Hansen M, et al. (2007) Lens epithelium-derived growth factor is an Hsp70-2 regulated guardian of lysosomal stability in human cancer. Cancer Res 67: 2559-2567.

11. Cohen B, Addadi Y, Sapoznik S, Meir G, Kalchenko V, et al. (2009) Transcriptional regulation of vascular endothelial growth factor C by oxidative and thermal stress is mediated by lens epithelium-derived growth factor/p75. Neoplasia 11: 921-933.
